# Patterns of viral infection in honey bee queens

**DOI:** 10.1099/vir.0.047019-0

**Published:** 2013-03

**Authors:** Roy Mathew Francis, Steen Lykke Nielsen, Per Kryger

**Affiliations:** Department of Agroecology, Science & Technology, Aarhus University, 4200 Slagelse, Denmark

## Abstract

The well-being of a colony and replenishment of the workers depends on a healthy queen. Diseases in queens are seldom reported, and our knowledge on viral infection in queens is limited. In this study, 86 honey bee queens were collected from beekeepers in Denmark. All queens were tested separately by two real-time PCRs: one for the presence of deformed wing virus (DWV), and one that would detect sequences of acute bee-paralysis virus, Kashmir bee virus and Israeli acute paralysis virus (AKI complex). Worker bees accompanying the queen were also analysed. The queens could be divided into three groups based on the level of infection in their head, thorax, ovary, intestines and spermatheca. Four queens exhibited egg-laying deficiency, but visually all queens appeared healthy. Viral infection was generally at a low level in terms of AKI copy numbers, with 134/430 tissues (31 %) showing the presence of viral infection ranging from 10^1^ to 10^5^ copies. For DWV, 361/340 tissues (84 %) showed presence of viral infection (DWV copies ranging from 10^2^ to 10^12^), with 50 tissues showing viral titres >10^7^ copies. For both AKI and DWV, the thorax was the most frequently infected tissue and the ovaries were the least frequently infected. Relative to total mass, the spermatheca showed significantly higher DWV titres than the other tissues. The ovaries had the lowest titre of DWV. No significant differences were found among tissues for AKI. A subsample of 14 queens yielded positive results for the presence of negative-sense RNA strands, thus demonstrating active virus replication in all tissues.

## Introduction

Honey bees (*Apis mellifera* L.) harbour a variety of pathogens such as bacteria, fungus, protozoa, viruses and pests such as mites and insects. Among these, viruses are the most recently characterized and least understood pathogens. Viruses in honey bees, once thought to be benign and asymptomatic infections (Bailey, 1967), have now been implicated as posing serious risks after the global spread of varroa mites (*Varroa destructor* Andersen & Trueman) carrying the viruses (Berthoud *et al.*, 2010; Carreck *et al.*, 2010; Martin *et al.*, 2012). Advancements in modern molecular methods and diagnostic techniques have led to the characterization of several viruses in honey bees (Chen & Siede, 2007).

Most virus studies focus on honey bee workers, as all non-reproductive functions are carried out by worker bees and heavy loss of worker bees can lead to the collapse of colonies (Vanengelsdorp *et al.*, 2009). Honey bee workers have relatively short lives and are susceptible to viral infections, occasionally showing overt outbreaks of disease. In contrast, the queen seldom shows symptomatic disease, although she is the longest-living member (3–4 years) of a bee hive (Winston, 1987). The queen is the main reproductive female in the bee colony (Visscher, 1989), and therefore the growth of a new colony and the replenishment of old workers depend on an egg-laying queen. One of the most extensive descriptions of diseases in queens was compiled in 1964 (Fyg, 1964). A few studies have explored viral infection in queens (Chen *et al.*, 2005a; de Miranda & Fries, 2008; Fievet *et al.*, 2006; Gauthier *et al.*, 2011). A study on honey bee queens by Chen *et al.* (2005a) showed that 93 % of the queens (*n* = 29) harboured multiple viral infections. Viruses tested include black queen cell virus (BQCV; 85 % of queens tested positive), chronic bee-paralysis virus (CBPV; 14 %), deformed wing virus (DWV; 100 %), Kashmir bee virus (KBV; 21 %) and sacbrood virus (SBV; 62 %), but acute bee-paralysis virus (ABPV) was absent. Queens are replaced by beekeepers for a variety of reasons including poor egg-laying capacity, aggressive colony behaviour and supersedure, but replacement due to disease is seldom reported (Tarpy *et al.*, 2012).

Our knowledge of viral infection in queens is rather limited; however, for some viruses, venereal transmission has been suggested (de Miranda & Fries, 2008; Yue *et al.*, 2007). The presence of viral particles in the seminal vesicles and mucus glands of young drones has been shown by transmission electron microscopy (Da Cruz-Landim *et al.*, 2012). The viruses analysed in this study were those of the ABPV–KBV–Israeli acute paralysis virus (IAPV) complex (AKI) (de Miranda *et al.*, 2010) and DWV (de Miranda & Genersch, 2010). These viruses were chosen because of their pathogenic importance, implications in global pandemics such as colony collapse disorder, association with varroa mites and widespread presence in bee colonies (Nordstrom *et al.*, 1999; Tentcheva *et al.*, 2004a).

DWV (Lanzi *et al.*, 2006) is the most common and widely studied honey bee virus and causes symptomatic crippled-wing syndrome (Möckel *et al.*, 2011), often seen in heavily infected bees and usually resulting in a reduced lifespan (Dainat *et al.*, 2012). DWV has been found in all developmental stages including the egg (Chen *et al.*, 2005b), larvae, pupae (Gauthier *et al.*, 2007), adults and all castes including queens (Chen *et al.*, 2005a; Gauthier *et al.*, 2011), workers and drones (Chen *et al.*, 2004b; Yanez *et al.*, 2012). DWV has been shown to be transmitted horizontally (Chen *et al.*, 2006a; Möckel *et al.*, 2011) from nurse bees to larvae and from infected semen to queen offspring by artificial insemination (de Miranda & Fries, 2008). DWV is known to be transmitted by varroa mites and is strongly correlated with mite infestation levels in bee colonies (Bowen-Walker *et al.*, 1999). DWV has been found to infect the head of queens, adipose tissues, gut, ovaries (Fievet *et al.*, 2006) and workers’ brains (Shah *et al.*, 2009). DWV has been reported in drone tissues (Fievet *et al.*, 2006), including semen (Yue *et al.*, 2006), suggesting that queens may be infected during mating. Both positive- and negative-strand DWV RNA has been found in the head, thorax and abdomen of crippled workers, but was found only in the thorax and abdomen of asymptomatic bees (Yue & Genersch, 2005). DWV has been reported to replicate in varroa mites based on the detection of negative-sense RNA in the mites (Gisder *et al.*, 2009; Ongus *et al.*, 2004). Other studies probing specific mite tissues have reported the absence of DWV replication in mites (Santillan-Galicia *et al.*, 2010; Zhang *et al.*, 2007).

ABPV, KBV and IAPV are three closely related virus species with a worldwide distribution (Ellis & Munn, 2005), commonly existing as covert low-titre infections. High titres of ABPV and IAPV on injection are reported to produce observable symptoms such as paralysis, trembling, inability to fly, and darkening and loss of hair from the thorax and abdomen (Bailey & Gibbs, 1964; Bailey *et al.*, 1963; Maori *et al.*, 2007; Ribiere *et al.*, 2008). These symptoms are usually not observed at the colony level, as the high-titre individuals are rapidly killed. However, a sharp decline in the adult population can be observed with severe infection (Todd *et al.*, 2004).

ABPV is one of most common honey bee viruses in Europe (Bakonyi *et al.*, 2002; Gauthier *et al.*, 2007; Nielsen *et al.*, 2008; Tentcheva *et al.*, 2004a) and has been implicated in winter losses (Siede *et al.*, 2008). The virus has been detected in the brain and hypopharyngeal glands (Bailey & Milne, 1969) and in faeces implying oral transmission (Chen *et al.*, 2006a). ABPV has been detected in semen indicating possible vertical transmission (Yue *et al.*, 2006). It has been shown to be vectored by mites (Ball, 1983, 1985) and implicated in varroa-associated colony losses (Békési *et al.*, 1999; Berényi *et al.*, 2006; Faucon *et al.*, 1992). KBV was first identified in *Apis cerana* bees from northern India (Bailey & Woods, 1977). KBV is serologically and genetically similar to ABPV, but the capsid protein profiles were reported to be different (Allen and Ball, 1995). KBV is generally less prevalent than ABPV (Nielsen *et al.*, 2008; Siede *et al.*, 2005; Tentcheva *et al.*, 2004a) but has been detected in workers, queens, honey, pollen, royal jelly and brood food (Shen *et al.*, 2005a). It has been found in the faeces of workers and queens (Hung, 2000), and was detected in eggs but not in queens (Chen *et al.*, 2006b). KBV seems to be the most virulent of all known honey bee viruses (Bailey *et al.*, 1979). KBV was detected in varroa mites (Hung & Shimanuki, 1999; Shen *et al.*, 2005b), and the mites were later found to be an effective vector (Chen *et al.*, 2004a) linked to colony losses (Hung *et al.*, 1995, 1996; Todd *et al.*, 2007).

IAPV is the most recently characterized of these three viruses (Maori *et al.*, 2007) and has been strongly implicated in colony collapse disorder (Cox-Foster *et al.*, 2007). Other studies have also associated IAPV with collapsing colonies (Antúnez *et al.*, 2006; Blanchard *et al.*, 2008). IAPV has been shown to be vectored by varroa mites (Di Prisco *et al.*, 2011). Transmission of IAPV has not been studied in much detail. All three of these viruses have been found in Denmark (Francis & Kryger, 2012). In this study, we investigated the qualitative and quantitative aspects of the AKI complex and DWV infection in five tissues of 86 honey bee queens.

## Results

Of the 86 queens analysed, seven were obtained from experimental hives with a heavy infestation of varroa mites, whilst 79 were obtained from beekeepers who had exchanged them for various reasons. Four queens were removed due to egg-laying deficiency, nine due to disease in the colony and seven due to old age, whilst 59 were considered healthy but were exchanged for breeding purposes. Visual examination during dissection revealed no obvious health deficiencies in any of the queens. Six queens (11Q21, 11Q34, 11Q35, 11Q48, 11Q49 and 11Q52) showed slight yellowish discoloration in the ovaries. However, we observed no significant relationship between this discoloration and queen removal due to poor egg laying or old age. The discoloured queens also showed no correlation with viral titres or titre groups. Six samples for which the β-actin assay failed were excluded for AKI and DWV. However, RNA in most of the samples was confirmed to be intact and detectable. For quantitative analysis, only results below a cut-off cycle threshold (*C*_t_) value of 34 were used, yielding quantitative results from 56 AKI, 510 β-actin and 341 DWV reactions.

### Viral prevalence

**Fig. 1.**
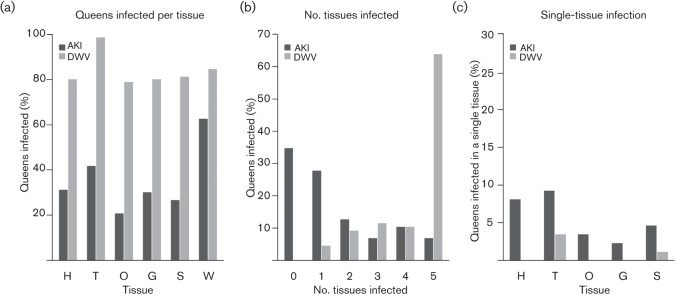
Qualitative results for 86 queens (and their workers) showing AKI and DWV infection. (a) Percentage of queens infected for each tissue with AKI and DWV. H, Head; T, thorax; O, ovary; G, gut; S, spermatheca; W, workers (workers were pooled and 30–40 mg was used). (b) Percentage of queens infected in 0, 1, 2, 3, 4 or 5 tissues. (c) Percentage of queens where only a single tissue was infected.

Of the 86 queens analysed, 56 were infected with AKI in at least one tissue, and among these, 41 queens had infected workers. Eighteen queens (21 %) were infected in the ovary for AKI (Fig. 1a). Only six (7 %) queens had all five tissues infected with AKI (Fig. 1b). The number of queens where only a single tissue was infected is shown in Fig. 1(c). In 13 AKI cases, the workers were infected but the respective queens were free of viruses in all tissues. For DWV, all 86 queens were infected in at least one tissue and 73 queens had DWV-infected workers. Eighty-five queens (99 %) were infected in the thorax. Fifty-five queens (64 %) had all five tissues infected with DWV.

### Viral titres

Single symptomatic adult worker bees with deformed wings were analysed individually as a measure to assess the viral titre in bees with obvious disease symptoms. These DWV-positive controls were then related to the DWV titres obtained in the queen tissues. The symptomatic adult workers showed a mean DWV viral load of 10^10^–10^12^ copies per bee (*n* = 11). Viral copies per adult crippled bee from other studies include 10^5^–10^8^ copies (Tentcheva *et al.*, 2004b), 10^5^–10^9^ copies (Gauthier *et al.*, 2007) and 10^10^–10^12^ copies (Gisder *et al.*, 2009).

The mean DWV titre across all 430 tissues in the 86 queens was 2×10^10^ copies (range 0–2×10^12^) whilst the mean number of AKI copies was 2×10^3^ (range 0–4×10^5^) (Fig. S1, available in JGV Online). The mean number of β-actin copies per tissue ranged from 7.4×10^7^ in the spermatheca to 2.8×10^9^ in the ovaries. In general, the AKI infection level was low and any notable infection could only be observed in the workers. The total number of copies per worker was not estimated, as the workers accompanying the queen were too few in number to be considered a true indicator of colony-level infection.

### Normalized viral titre

The tissue weights of three queens were measured to normalize the quantitative copies against tissue weight. The total weight of the queens (*n* = 3) was 184±13 mg. The weights of queen tissues (*n* = 3) were as follows: head, 11.5±1.8 mg; thorax, 54.5±4.7 mg; ovary, 42.5±11.1 mg; gut, 13.1±2.2 mg; and spermatheca, 0.9±0.1 mg. The total number of copies per tissue was divided by the respective weights of each tissue in grams to obtain copies (g tissue)^−1^. For AKI, the difference in titres between tissues was not significant. For DWV, a non-parametric Friedman test showed viral titres between the five tissues to be significantly different (*P*<2.2×10^−16^). A post-hoc Wilcoxon test for paired data using Bonferroni correction revealed the following results: significant differences (*P*<0.05) were found between the following tissue pairs: head–ovaries, head–spermatheca, thorax–ovaries, thorax–spermatheca, gut–ovaries, ovaries–spermatheca and gut–spermatheca.

**Fig. 2.**
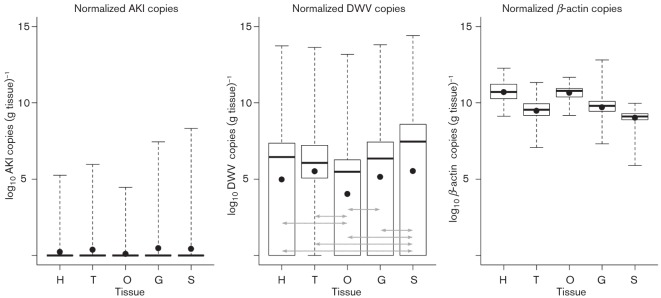
Normalized copies [copies (g tissue)^−1^] of AKI, DWV and β-actin in various queen tissues. The *x*-axis shows the different tissues (see Fig. 1 for abbreviations), whilst the *y*-axis shows log_10_ normalized copies. The box and whisker plots show range, inter-quartile range and median (thick horizontal bar). The black dot denotes the mean. Horizontal lines with arrowheads indicate tissues where significant differences were observed based on a Wilcoxon paired test.

Fig. 2 shows normalized copies [copies (g tissue)^−1^] of AKI, DWV and β-actin among the different tissues. Both AKI and DWV show the highest mean number of viral copies in the spermatheca, whilst the ovary was the least infected (not significant for AKI). The spermatheca was the most infected tissue for AKI in six queens (7 %) and for DWV in 48 queens (56 %). The thorax was the most infected tissue for DWV in nine queens (10 %).

### Hierarchial clustering

**Fig. 3.**
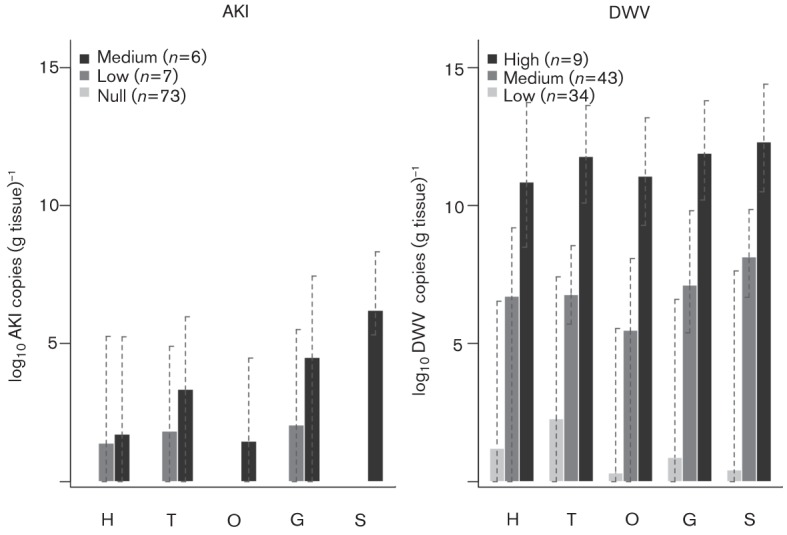
Mean number of viral copies in the three infection groups for AKI and DWV defined by hierarchical clustering on normalized copies. The error bars show the range. For AKI, the null group was completely zero (no AKI copies), whilst the low- and medium-titre groups were not very different, possibly due to the small number of positive AKI samples. The DWV copies showed very clear distinctions into high-, medium- and low-titre groups.

The hierarchical clustering did not reveal any evidence of viral progression through the tissues examined. However, the queens were grouped based on AKI and DWV viral titres across all tissues (Fig. S2). For both viruses, we detected three obvious clusters; however, these were more linked to the overall level of viral titres than to specific tissues being targeted by viral infection. Fig. 3 shows the viral titres for the different tissues in the three groups. The AKI clustering showed the low-level infection group to be identical, as they all had zero AKI copies. The percentages of queens in the three AKI clusters were 85 % in the zero-titre group, 8 % in the low-titre group and 7 % in the medium-titre group. The viral copies in the three AKI groups for all tissues were as follows: zero titre, 0–3.1×10^5^ copies in the low-titre group and 0–2×10^8^ copies in the medium-titre group. The percentages of queens in the three DWV clusters were 40 % in the low-titre group, 50 % in the medium-titre group and 10 % in the high-titre group, and the range of viral copies in the three groups were 0–4.2×10^7^ copies in the low-titre group, 0–7.1×10^9^ in the medium-titre group and 3×10^8^–2.5×10^14^ copies in the high-titre group. Therefore, only 7–10 % of the queens showed detrimental infection levels.

### Strand-specific analysis

**Fig. 4.**
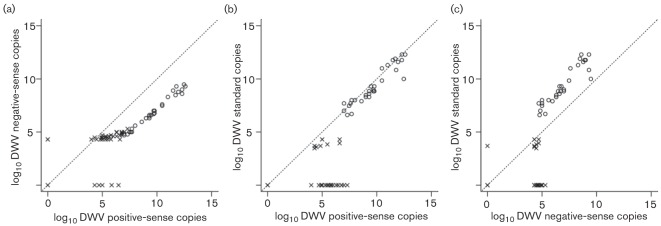
Correlation between DWV titres in all tissues for strand-specific primers and standard primers in eight high-titre queens (○) and six low-titre queens (×). (a) All high-titre queens showed more copies of positive-sense than negative-sense RNA. The low-titre queens did not show a linear relationship. (b) The positive-sense-specific primers yielded higher efficiency than standard primers in most high-titre queens. (c) The standard primers mostly detected positive-sense RNA. In (b) and (c), the low-titre queens did not yield consistent results between the methods used, possibly due to quantification limitation or false priming.

For DWV, strand-specific analysis was carried out on six high-titre queens and eight low-titre queens to analyse the quantity of positive- and negative-sense DWV copies in five tissues. A correlation analysis was carried out with DWV positive-sense copies, DWV negative-sense copies and standard DWV copies (Fig. 4). DWV positive-sense copies were positively correlated with negative strands (*R*^2^ = 0.77). The standard DWV results were positively correlated with positive-sense DWV copies (*R*^2^ = 0.82) and with negative-sense DWV copies (*R*^2^ = 0.72). As shown in Table S1, the number of positive-sense copies exceed the number of negative-sense copies and also the standard DWV copies. Some queens that yielded negative results using the standard primers were found to be quantifiable using strand-specific positive-sense primers (*n* = 26) and negative-sense primers (*n* = 25). Negative-strand detection was not carried out for AKI due to its low prevalence in the queens studied here.

## Discussion

In this study, we demonstrated the presence of AKI and DWV in five queen tissues: head, thorax, ovary, gut and spermatheca, as well as in accompanying workers. AKI and DWV were chosen due to their impact on colony health and widespread prevalence. BQCV, a virus commonly associated with queens, was not studied here as it is rarely found in Denmark (Nielsen *et al.*, 2008). The head is commonly used in viral diagnosis, and viral presence in the head is thought to be an indicator of high-tire infection. The thorax was chosen, as high DWV titres in workers often result in deformed wings. The gut often shows the presence of ingested viruses and is indicative of transmission by trophallaxis. The ovaries and spermatheca were selected to investigate venereal transmission.

DWV copies in the 86 queens tested ranged from 0 to 2×10^12^ copies compared with 3.2×10^7^ copies (Fievet *et al.*, 2006) and 10^8^–10^12^ copies (Gauthier *et al.*, 2011) in previous studies. The AKI titres ranged from 0 to 4×10^5^ copies, and none of the 56 AKI-positive queens showed titres close to those of symptomatic workers (10^10^ copies). Given the low prevalence and low titres of AKI, we decided not to investigate which of the three viruses of this complex was actually present. Detection of high AKI titres is unlikely in live queens, as AKI would kill them rapidly.

The queens were generally found to be in a healthy state, supporting the beekeepers’ observations (Table S2). In queens with high viral titres, the accompanying workers also exhibited equal or higher viral titres. Visual examination of the queens showed no obvious disease symptoms in most queens, as reported previously (Chen *et al.*, 2005b). Six queens showed a slight yellowish discoloration in the ovaries. The level of discoloration was far less than the discoloration symptoms observed previously (Gauthier *et al.*, 2011), and we found no correlation between discoloration and viral load. The detailed study by Gauthier *et al.* (2011) focused on a novel pathology affecting queen ovaries characterized by yellow discoloration and degenerative lesions in the follicles. The Gauthier study also compared viral infection in virgin queens with that in mated queens. The mated queens (*n* = 30, from across France) showed the presence of DWV (100 %) and IAPV (10 %) but absence of ABPV and KBV. The virgin queens (*n* = 40) showed a lower presence of DWV (37 %) and none of the AKI viruses was detected. This indicated that mating can transmit viral disease; however, mated queens are older than virgin queens, which results in a higher probability of infection from workers.

Nine queens showed DWV titres close to the level of deformed-wing workers, but none of these queens exhibited any obvious signs of disease. Incidences of crippling overt infection in queens have seldom been reported (Williams *et al.*, 2009). Queens are unlikely to be exposed to the virus during the developmental stages, as queen cells are not attractive to varroa mites. Trophallaxis or vector-mediated pathways seem probable routes of infection in adult queens, as they are constantly fed and consume twice their own weight each day. The ovaries had the lowest infection counts and the lowest viral titres. Hence, it is possible that the ovaries may be protected to preserve the vital reproductive functions.

Honey bee queens are known to mate with more than ten drones (Schluns *et al.*, 2005). Each mating increases the risk to the queen, as it has been shown that many DWV-infected drones arrive at the drone congregation areas for mating (Yanez *et al.*, 2012). The presence of DWV has been demonstrated in multiple tissues of drones (Fievet *et al.*, 2006), including drone semen (Yue *et al.*, 2006) and the endophallus, which remains in the queen after mating with high viral titres of up to 10^9^ copies (Yanez *et al.*, 2012). It is difficult to conclude from the previous studies of queen viral infection (Chen *et al.*, 2005a; Gauthier *et al.*, 2011) whether the higher prevalence of DWV in mated queens results from infection during mating (de Miranda & Fries, 2008) or simply because mated queens are older than virgin queens.

A recent publication (Martin *et al.*, 2012) demonstrating a selective sweep of DWV following the introduction of varroa mites could be the reason for the widespread infection of queens. Part of the virus population seems more adapted to vector transmission than to venereal transmission. Assuming that venereal transmission is the main source of queen infection, we would have expected the spermatheca to be the most frequently infected tissue. Furthermore, we would have expected the infection to be confined to the spermatheca in order to avoid killing the queen. In contrast, our results indicated that the thorax is the most frequently infected part of the queen. The highest DWV titres were actually observed in the spermatheca, based on normalized data. However, it should be noted that the small tissue size of the spermatheca is coupled with uncertainty of weight measurements and sperm content. The debate regarding the polyandrous mating system (Kraus & Moritz, 2010) of honey bees centres on the individual cost of the queen relative to the colony-level benefits from increased genetic diversity (Mattila & Seeley, 2007). Whilst we cannot exclude venereal transmission, it seems to play a minor role in queen viral titres, and thus the cost of multiple mating to the queen seems low.

In the current study, the spermatheca showed the highest titre of DWV whereas the ovaries had the lowest. This suggests that DWV transmission during mating is confined mainly to the spermatheca, in spite of the deposition of sperm in the oviduct following mating. Only a minor difference was observed in the fraction of queens with infected ovaries compared with those with infected spermatheca. Of the queens with DWV in either the ovaries or the spermatheca (*n* = 55), 54 queens had higher viral titres in the spermatheca than the ovary (Table S3). The question of venereal transmission has been addressed by means of artificial insemination of honey bee queens with semen from infected drones (de Miranda & Fries, 2008), demonstrating the transmission of DWV in two of three inseminated queens, thus indicating a venereal route, although this may not be 100 % effective. The DWV titres in the ovaries were higher than those of the spermatheca in the two queens where transmission was successful, in contrast to our finding. We failed to detect any tissue preference for either virus using hierarchical clustering. The low-level infection queens showed low viral titres in all tissues, and high titres were observed in all tissues of high-level infection queens.

The presence of negative-sense RNA indicates active replication of virus in the particular tissue, rather than a passive presence. Several tissues negative with the standard DWV primers were found to be quantifiable with strand-specific primers. This discrepancy could result from the use of random hexamers for cDNA conversion in the standard methodology, which leads to competition between bee RNA and viral RNA. In contrast, strand-specific primers used for cDNA conversion transcribe viral RNA more selectively and efficiently. Indeed, higher titres of positive-strand-specific copies were evident for most queens (Fig. 4b). However, for the low-titre queens, there was considerable variance between the results from the standard primers and the negative- and positive-strand-specific primers, either because viral titres in these queens were close to the limit of quantification or because of false positives (Boncristiani *et al.*, 2009). In Fig. 4(a), it can be observed that, for the low-titre queens, several queens were quantifiable using either of the strand-specific primers, but the linear relationship only started at 10^7^ positive-sense copies. This was probably due to false priming, as is also evident from Fig. 4(b) and (c). Thus, queens that lie outside the linear range of copies (<10^7^) may not be quantified accurately using strand-specific primers. From Fig. 4(c), it can be seen that, for the high-titre queens, there were 1000 times more DWV standard copies compared with DWV strand-specific copies. Several low-titre queens yielded ambiguous temperature profiles based on dissociation curves. From all of this, we infer limitations for the quantification of negative-sense copies in low-titre queens.

In summary, our results suggest covert viral infection in queens from most healthy colonies. In colonies with severe varroa mite infestation, the queen may eventually be infected. Thus, queens are not entirely immune to viral infections, which appear to be transmitted from their worker offspring rather than via the often-discussed sexual transmission during multiple mating. The protective mechanisms behind this pattern remain to be elucidated.

## Methods

### 

#### Sample collection and processing.

A total of 86 honey bee queens of various ages were analysed in this study. Seventy-nine queens were donated by Danish beekeepers. Observations by the beekeepers leading to queen exchange were collected and recorded if possible. The queens were placed in queen cages along with a few workers and mailed alive to the laboratory. Seven queens were collected from experimental colonies that were known to be heavily infested with varroa mites.

Upon arrival, the queen and workers from each cage were processed as separate samples, but the accompanying workers from each queen were pooled. The queens and workers were killed with CO_2_ gas. The queen was pinned onto a sterile dissection surface with clean pins. Dissection was carried out using sterile scissors and forceps cleaned with 70 % alcohol between subsamples. Each queen and her individual organs were inspected visually for obvious signs of infection or disease. Each queen’s organs were dissected and stored as seven separate subsamples: head, two thoracic (left and right), two ovarial (left and right), intestines and spermatheca. Dissected tissues from the queens were stored in 1.5 ml microcentrifuge tubes and workers were stored in 50 ml plastic bottles with tight-fitting screw caps. Samples were freeze dried on a Heto LyoPro 6000 apparatus for ~72 h at a pressure of 0.05 hPa and at −80 °C. After lyophilization, all samples were stored immediately at −80 °C until further use.

#### RNA extraction and quantitative PCR (qPCR).

Dissected queen tissues were flash frozen using liquid N_2_ and crushed using micropestles. Worker samples were homogenized on a Geno/Grinder 2000 apparatus for 1 min at 1500 r.p.m. with metal beads added to the bottle. For pooled workers, a small amount of tissue (30–40 mg) was used. Total RNA was extracted using a Nucleospin RNA II kit (Macherey-Nagel). Extracted RNA (60 µl) was stored in 96-well microtitre plates (Thermo Scientific) at −80 °C until further use. The RNA was transcribed into cDNA using a High-Capacity cDNA Reverse Transcription kit (Applied Biosystems). RNA (10 µl) was added to 10 µl cDNA master mix yielding a 20 µl cDNA solution. The incubation conditions were as recommended by the kit: 10 min at 25 °C, 120 min at 37 °C and 5 min at 85 °C. The cDNA solution was then diluted tenfold (10 µl cDNA in 90 µl sterile H_2_O) and stored at −80 °C.

Real-time qPCR assays were carried out on an ABI PRISM 7600HT (Applied Biosystems) using SYBR Green DNA binding dye (Applied Biosystems). The primers used in this study are listed in Table S4. Two sets of primers were used for each virus (AKI and DWV), referred to as outer and inner primers as in Gauthier *et al.* (2007). The AKI viruses were detected in a single assay using a single pair of primers referred to as ‘AKI’ primers (Francis & Kryger, 2012). The volume for qPCRs was 12 µl, with a final primer concentration of 0.4 µM. Diluted (tenfold) cDNA was used in constructing the standard curve and RNase-free water was used as template for the negative controls. All reactions were run on optical 384-well PCR plates in replicates of two. Standard cycling parameters were used for thermal cycling and the dissociation curve. Twelve symptomatic bees with crippled wings were collected and processed similarly to estimate viral load in DWV symptomatic bees.

#### Data processing and analysis.

Nine dilutions for the AKI and β-actin4 primers and eight dilutions for the DWV3 primers were used to create standard curves and subsequent linear regression (Fig. S3). The reaction efficiencies for AKI, β-actin4 and DWV3 were 2.04, 2.04 and 1.98, respectively. Pearson’s correlation coefficients (*C*_t_ values against log_10_ of copies) for AKI, β-actin and DWV were −0.998, −1 and −0.998, respectively. The baseline was automatically set and a manual threshold of 0.19 was used for all control runs and test runs. Dissociation profiles for all reactions were examined visually and flagged as appropriate. Data from the qPCR runs were analysed in r (R Development Core Team, 2011) and Microsoft Excel. Replicates showing a coefficient of variation of >10 % were flagged, and replicates were examined and corrected manually if required. Samples that did not cross the threshold before cycle 40 were given a *C*_t_ value of 0 (no virus). Samples with an incorrect melting-curve profile were given a *C*_t_ value of 0.

Whilst converting to copy numbers, copies near 1 were rounded to 0 or 1, as it is assumed that there has to be one copy of the virus/β-actin gene or none. As thorax and ovary samples were split and processed separately, the best *C*_t_ values of the biological replicates were selected, converted to copies and then multiplied by 2. Based on the standard curves, a *C*_t_ value of 34 was chosen as a cut-off for quantitative results because the standard curves were no longer linear after cycle 34. All results where fluorescence crossed the threshold after cycle 34 were considered qualitatively positive but not quantifiable and were omitted from quantitative calculations. These were, however, included in the qualitative calculations. Based on the regression, cycle 34 corresponded to 13, 10 and 243 copies of AKI, β-actin and DWV, respectively.

#### Normalization to tissue weight.

Three queens were dissected and their tissues weighed separately to determine individual tissue weights (Table S5). The absolute number of quantifiable copies per tissue was divided by the mean weight of the respective tissues to obtain viral/β-actin copies (g tissue)^−1^. These copies are referred to as normalized copies. This was carried out only on queen tissues and not on the accompanying workers, as the workers were pooled samples and not representative of colony-level worker titres. The normalized copies were used in further analyses. Hierarchical clustering was carried out to identify possible patterns in progression of the viral infection. This was also a means of categorizing the queens based on viral titres in all the tissues. For hierarchical clustering, Euclidean distances were computed for AKI and DWV copies separately and clustering (*K* = 3) was carried out on the distances using Ward’s method, based on the viral titres of all queen tissues.

#### Negative-strand analysis.

Strand-specific qPCR was performed on 14 selected queens (six high-titre queens and eight low-titre queens) to quantify DWV-positive and -negative strands across five tissues. The presence of the negative strand of DWV indicated active replication of the virus in the specific tissue. Each queen was analysed for five tissues (head, thorax, ovary, intestines and spermatheca). RNA (2 µl) was converted to cDNA in a 12 µl reaction using a ThermoScript Reverse Transcriptase kit (Invitrogen) with 10 mM dNTP mix (Invitrogen). The cDNA conversion was carried out in two separate reactions, one reaction containing a tagged DWV forward primer and the second containing a tagged DWV reverse primer (Table S1). The reaction was processed in 96-well plates and placed on a 2720 Thermal Cycler (Applied Biosystems) with the following temperature profile: 60 °C for 55 min, followed by 85 °C for 5 min. The unique nature of the high-temperature-tolerant reverse transcriptase allowed reverse transcription to be carried out at a high temperature to ensure high-specificity primer binding. The qPCR was carried out using the tag and one of the DWV3 primers. Non-enzyme controls and non-template controls were included between steps. Similar qPCR volumes and conditions were used for DWV as indicated above and the same standard curves were used to estimate the number of viral copies µl^−1^. The same *C*_t_ cut-off value of 34 was used to define quantitative copies within the range of standard curves. The number of viral copies µl^−1^ was converted to viral copies per tissue, followed by normalizing to tissue weights.

## References

[r1] Allen M. F. Ball B. V. **(**1995**).** Characterisation and serological relationships of strains of Kashmir bee virus. Ann Appl Biol 126, 471–484 10.1111/j.1744-7348.1995.tb05382.x

[r2] Antúnez K. D’Alessandro B. Corbella E. Ramallo G. Zunino P. **(**2006**).** Honeybee viruses in Uruguay. J Invertebr Pathol 93, 67–70 10.1016/j.jip.2006.05.009 16843485

[r3] Bailey L. **(**1967**).** The incidence of virus diseases in the honey bee. Ann Appl Biol 60, 43–48 10.1111/j.1744-7348.1967.tb05920.x 6076167

[r4] Bailey L. Gibbs A. J. **(**1964**).** Acute infection of bees with paralysis virus. J Insect Pathol 6, 395–407

[r5] Bailey L. Milne R. G. **(**1969**).** The multiplication regions and interaction of acute and chronic bee-paralysis viruses in adult honey bees. J Gen Virol 4, 9–14 10.1099/0022-1317-4-1-9

[r6] Bailey L. Woods R. D. **(**1977**).** Two more small RNA viruses from honey bees and further observations on sacbrood and acute bee-paralysis viruses. J Gen Virol 37, 175–182 10.1099/0022-1317-37-1-175

[r7] Bailey L. Gibbs A. J. Woods R. D. **(**1963**).** Two viruses from adult honey bees (*Apis* *mellifera* Linnaeus). Virology 21, 390–395 10.1016/0042-6822(63)90200-9 14081363

[r8] Bailey L. Carpenter J. M. Woods R. D. **(**1979**).** Egypt bee virus and Australian isolates of Kashmir bee virus. J Gen Virol 43, 641–647 10.1099/0022-1317-43-3-641

[r9] Bakonyi T. Farkas R. Szendroi A. Dobos-Kovacs M. Rusvai M. **(**2002**).** Detection of acute bee paralysis virus by RT-PCR in honey bee and *Varroa destructor* field samples: rapid screening of representative Hungarian apiaries. Apidologie (Celle) 33, 63–74 10.1051/apido:2001004

[r10] Ball B. V. **(**1983**).** The association of *Varroa jacobsoni* with virus diseases of honey bees. Exp Appl Acarol 19, 607–613

[r11] Ball B. V. **(**1985**).** Acute paralysis virus isolates from honeybee colonies infested with *Varroa* *jacobsoni*. J Apic Res 24, 115–119

[r12] Békési L. Ball B. V. Dobos-Kovács M. Bakonyi T. Rusvai M. **(**1999**).** Occurrence of acute paralysis virus of the honey bee (*Apis mellifera*) in a Hungarian apiary infested with the parasitic mite *Varroa jacobsoni*. Acta Vet Hung 47, 319–324 10.1556/AVet.47.1999.3.5 10497825

[r13] Berényi O. Bakonyi T. Derakhshifar I. Köglberger H. Nowotny N. **(**2006**).** Occurrence of six honeybee viruses in diseased Austrian apiaries. Appl Environ Microbiol 72, 2414–2420 10.1128/AEM.72.4.2414-2420.2006 16597939 PMC1449027

[r14] Berthoud H. Imdorf A. Haueter M. Radloff S. Neumann P. **(**2010**).** Virus infections and winter losses of honey bee colonies (*Apis mellifera*). J Apic Res 49, 60–65 10.3896/IBRA.1.49.1.08

[r15] Blanchard P. Schurr F. Celle O. Cougoule N. Drajnudel P. Thiéry R. Faucon J. P. Ribière M. **(**2008**).** First detection of Israeli acute paralysis virus (IAPV) in France, a dicistrovirus affecting honeybees (*Apis mellifera*). J Invertebr Pathol 99, 348–350 10.1016/j.jip.2008.07.006 18703069

[r16] Boncristiani H. F. Jr Di Prisco G. Pettis J. S. Hamilton M. Chen Y. P. **(**2009**).** Molecular approaches to the analysis of deformed wing virus replication and pathogenesis in the honey bee, *Apis mellifera.* Virol J 6, 221 10.1186/1743-422X-6-221 20003360 PMC2797523

[r17] Bowen-Walker P. L. Martin S. J. Gunn A. **(**1999**).** The transmission of deformed wing virus between honeybees (*Apis mellifera* L.) by the ectoparasitic mite *Varroa jacobsoni* Oud. J Invertebr Pathol 73, 101–106 10.1006/jipa.1998.4807 9878295

[r18] Carreck N. L. Ball B. V. Martin S. **(**2010**).** Honey bee colony collapse and changes in viral prevalence associated with *Varroa destructor*. J Apic Res 49, 93–94 10.3896/IBRA.1.49.1.13

[r19] Chen Y. P. Siede R. **(**2007**).** Honey bee viruses. Adv Virus Res 70, 33–80 10.1016/S0065-3527(07)70002-7 17765703

[r20] Chen Y. P. Pettis J. S. Evans J. D. Kramer M. Feldlaufer M. F. **(**2004a**).** Transmission of Kashmir bee virus by the ectoparasitic mite *Varroa destructor*. Apidologie (Celle) 35, 441–448 10.1051/apido:2004031

[r21] Chen Y. P. Zhao Y. Hammond J. Hsu H.-T. Evans J. Feldlaufer M. **(**2004b**).** Multiple virus infections in the honey bee and genome divergence of honey bee viruses. J Invertebr Pathol 87, 84–93 10.1016/j.jip.2004.07.005 15579317

[r22] Chen Y. P. Pettis J. S. Feldlaufer M. F. **(**2005a**).** Detection of multiple viruses in queens of the honey bee *Apis mellifera* L. J Invertebr Pathol 90, 118–121 10.1016/j.jip.2005.08.005 16214161

[r23] Chen Y. P. Higgins J. A. Feldlaufer M. F. **(**2005b**).** Quantitative real-time reverse transcription-PCR analysis of deformed wing virus infection in the honeybee (*Apis mellifera* L.). Appl Environ Microbiol 71, 436–441 10.1128/AEM.71.1.436-441.2005 15640219 PMC544241

[r24] Chen Y. P. Evans J. Feldlaufer M. **(**2006a**).** Horizontal and vertical transmission of viruses in the honey bee, *Apis mellifera*. J Invertebr Pathol 92, 152–159 10.1016/j.jip.2006.03.010 16793058

[r25] Chen Y. P. Pettis J. S. Collins A. Feldlaufer M. F. **(**2006b**).** Prevalence and transmission of honeybee viruses. Appl Environ Microbiol 72, 606–611 10.1128/AEM.72.1.606-611.2006 16391097 PMC1352288

[r26] Cox-Foster D. L. Conlan S. Holmes E. C. Palacios G. Evans J. D. Moran N. A. Quan P. L. Briese T. Hornig M. **& other authors (**2007**).** A metagenomic survey of microbes in honey bee colony collapse disorder. Science 318, 283–287 10.1126/science.1146498 17823314

[r27] Da Cruz-Landim C. Roat T. C. Fernadez F. C. **(**2012**).** Virus present in the reproductive tract of asymptomatic drones of honey bee (*Apis mellifera* l.), and possible infection of queen during mating. Microsc Res Tech 75, 986–990 10.1002/jemt.22024 22419610

[r28] Dainat B. Evans J. D. Chen Y. P. Gauthier L. Neumann P. **(**2012**).** Dead or alive: deformed wing virus and *Varroa destructor* reduce the life span of winter honeybees. Appl Environ Microbiol 78, 981–987 10.1128/AEM.06537-11 22179240 PMC3273028

[r29] de Miranda J. R. Fries I. **(**2008**).** Venereal and vertical transmission of deformed wing virus in honeybees (*Apis mellifera* L.). J Invertebr Pathol 98, 184–189 10.1016/j.jip.2008.02.004 18358488

[r30] de Miranda J. R. Genersch E. **(**2010**).** Deformed wing virus. J Invertebr Pathol 103 (Suppl. 1), S48–S61 10.1016/j.jip.2009.06.012 19909976

[r31] de Miranda J. R. Cordoni G. Budge G. **(**2010**).** The acute bee paralysis virus–Kashmir bee virus–Israeli acute paralysis virus complex. J Invertebr Pathol 103 (Suppl. 1), S30–S47 10.1016/j.jip.2009.06.014 19909972

[r32] Di Prisco G. Pennacchio F. Caprio E. Boncristiani H. F. Jr Evans J. D. Chen Y. **(**2011**).** *Varroa destructor* is an effective vector of Israeli acute paralysis virus in the honeybee, *Apis mellifera*. J Gen Virol 92, 151–155 10.1099/vir.0.023853-0 20926637

[r33] Ellis J. D. Munn P. A. **(**2005**).** The worldwide health status of honey bees. Bee World 86, 88–101

[r34] Faucon J. P. Vitu C. Russo P. Vignoni M. **(**1992**).** Diagnosis of acute paralysis – application to epidemic honeybee diseases in France during 1990. Apidologie (Celle) 23, 139–146 10.1051/apido:19920206

[r35] Fievet J. Tentcheva D. Gauthier L. de Miranda J. Cousserans F. Colin M. E. Bergoin M. **(**2006**).** Localization of deformed wing virus infection in queen and drone *Apis* *mellifera* L. Virol J 3, 16 10.1186/1743-422X-3-16 16569216 PMC1475838

[r36] Francis R. M. Kryger P. **(**2012**).** Single assay detection of acute bee paralysis virus, Kashmir bee virus and Israeli acute paralysis virus. J Apic Sci 56, 137–146

[r37] Fyg W. **(**1964**).** Anomalies and diseases of the queen honey bee. Annu Rev Entomol 9, 207–224 10.1146/annurev.en.09.010164.001231

[r38] Gauthier L. Tentcheva D. Tournaire M. Dainat B. Cousserans F. Colin M. E. Bergoin M. **(**2007**).** Viral load estimation in asymptomatic honey bee colonies using the quantitative RT-PCR technique. Apidologie (Celle) 38, 426–435 10.1051/apido:2007026

[r39] Gauthier L. Ravallec M. Tournaire M. Cousserans F. Bergoin M. Dainat B. de Miranda J. R. **(**2011**).** Viruses associated with ovarian degeneration in *Apis mellifera* L. queens. PLoS ONE 6, e16217 10.1371/journal.pone.0016217 21283547 PMC3026828

[r40] Gisder S. Aumeier P. Genersch E. **(**2009**).** Deformed wing virus: replication and viral load in mites (*Varroa destructor*). J Gen Virol 90, 463–467 10.1099/vir.0.005579-0 19141457

[r41] Hung A. C. F. **(**2000**).** PCR detection of Kashmir bee virus in honey bee excreta. J Apic Res 39, 103–106

[r42] Hung A. C. F. Shimanuki H. **(**1999**).** A scientific note on the detection of Kashmir bee virus in individual honeybees and *Varroa jacobsoni* mites. Apidologie (Celle) 30, 353–354 10.1051/apido:19990414

[r43] Hung A. C. F. Adams J. R. Shimanuki H. **(**1995**).** Bee parasitic mite syndrome. 2. The role of varroa mite and viruses. Am Bee J 135, 702–704

[r44] Hung A. C. F. Shimanuki H. Knox D. A. **(**1996**).** Inapparent infection of acute paralysis virus and Kashmir bee virus in the US honey bees. Am Bee J 136, 874–876

[r45] Kraus F. B. Moritz R. F. A. **(**2010**).** Extreme polyandry in social hymenoptera: evolutionary causes and consequences for colony organisation. In Animal Behaviour: Evolution and Mechanisms, pp. 413–439 Edited by Kappeler P. Berlin/Heidelberg: Springer 10.1007/978-3-642-02624-9_14

[r46] Lanzi G. de Miranda J. R. Boniotti M. B. Cameron C. E. Lavazza A. Capucci L. Camazine S. M. Rossi C. **(**2006**).** Molecular and biological characterization of deformed wing virus of honeybees (*Apis mellifera* L.). J Virol 80, 4998–5009 10.1128/JVI.80.10.4998-5009.2006 16641291 PMC1472076

[r47] Maori E. Lavi S. Mozes-Koch R. Gantman Y. Peretz Y. Edelbaum O. Tanne E. Sela I. **(**2007**).** Isolation and characterization of Israeli acute paralysis virus, a dicistrovirus affecting honeybees in Israel: evidence for diversity due to intra- and inter-species recombination. J Gen Virol 88, 3428–3438 10.1099/vir.0.83284-0 18024913

[r48] Martin S. J. Highfield A. C. Brettell L. Villalobos E. M. Budge G. E. Powell M. Nikaido S. Schroeder D. C. **(**2012**).** Global honey bee viral landscape altered by a parasitic mite. Science 336, 1304–1306 10.1126/science.1220941 22679096

[r49] Mattila H. R. Seeley T. D. **(**2007**).** Genetic diversity in honey bee colonies enhances productivity and fitness. Science 317, 362–364 10.1126/science.1143046 17641199

[r50] Möckel N. Gisder S. Genersch E. **(**2011**).** Horizontal transmission of deformed wing virus: pathological consequences in adult bees (*Apis mellifera*) depend on the transmission route. J Gen Virol 92, 370–377 10.1099/vir.0.025940-0 20965988

[r51] Nielsen S. L. Nicolaisen M. Kryger P. **(**2008**).** Incidence of acute bee paralysis virus, black queen cell virus, chronic bee paralysis virus, deformed wing virus, Kashmir bee virus and sacbrood virus in honey bees (*Apis mellifera*) in Denmark. Apidologie (Celle) 39, 310–314 10.1051/apido:2008007

[r52] Nordstrom S. Fries I. Aarhus A. Hansen H. Korpela S. **(**1999**).** Virus infections in Nordic honey bee colonies with no, low or severe *Varroa jacobsoni* infestations. Apidologie (Celle) 30, 475–484 10.1051/apido:19990602

[r53] Ongus J. R. Peters D. Bonmatin J. M. Bengsch E. Vlak J. M. van Oers M. M. **(**2004**).** Complete sequence of a picorna-like virus of the genus *Iflavirus* replicating in the mite *Varroa destructor*. J Gen Virol 85, 3747–3755 10.1099/vir.0.80470-0 15557248

[r54] R Development Core Team **(**2011**).** r: A Language and Environment for Statistical Computing. Vienna: R Foundation for Statistical Computing

[r55] Ribiere M. Ball B. V. Aubert M. **(**2008**).** Natural history and geographic distribution of honey bee viruses. In Virology and the Honey Bee, pp. 15–84 Edited by Aubert B. Ball A. Fries I. Milani N. Moritz R. F. A. Brussels: VIth Framework, European Commission

[r56] Santillan-Galicia M. T. Ball B. V. Clark S. J. Alderson P. G. **(**2010**).** Transmission of deformed wing virus and slow paralysis virus to adult bees (*Apis mellifera* L.) by *Varroa* *destructor*. J Apic Res 49, 141–148 10.3896/IBRA.1.49.2.01

[r57] Schluns H. Moritz R. F. A. Neumann P. Kryger P. Koeniger G. **(**2005**).** Multiple nuptial flights, sperm transfer and the evolution of extreme polyandry in honeybee queens. Anim Behav 70, 125–131 10.1016/j.anbehav.2004.11.005

[r58] Shah K. S. Evans E. C. Pizzorno M. C. **(**2009**).** Localization of deformed wing virus (DWV) in the brains of the honeybee, *Apis mellifera* Linnaeus. Virol J 6, 182 10.1186/1743-422X-6-182 19878557 PMC2779808

[r59] Shen M. Cui L. Ostiguy N. Cox-Foster D. **(**2005a**).** Intricate transmission routes and interactions between picorna-like viruses (Kashmir bee virus and sacbrood virus) with the honeybee host and the parasitic varroa mite. J Gen Virol 86, 2281–2289 10.1099/vir.0.80824-0 16033976

[r60] Shen M. Yang X. Cox-Foster D. Cui L. **(**2005b**).** The role of varroa mites in infections of Kashmir bee virus (KBV) and deformed wing virus (DWV) in honey bees. Virology 342, 141–149 10.1016/j.virol.2005.07.012 16109435

[r61] Siede R. Derakhshifar I. Otten C. Berenyi O. Bakonyi T. Kolberger H. Buchleir R. **(**2005**).** Prevalence of Kashmir bee virus in central Europe. J Apic Res 44, 129–129

[r62] Siede R. Konig M. Buchler R. Failing K. Thiel H. J. **(**2008**).** A real-time PCR based survey on acute bee paralysis virus in German bee colonies. Apidologie (Celle) 39, 650–661 10.1051/apido:2008044

[r63] Tarpy D. R. Keller J. J. Caren J. R. Delaney D. A. **(**2012**).** Assessing the mating ‘health’ of commercial honey bee queens. J Econ Entomol 105, 20–25 10.1603/EC11276 22420250

[r64] Tentcheva D. Gauthier L. Zappulla N. Dainat B. Cousserans F. Colin M. E. Bergoin M. **(**2004a**).** Prevalence and seasonal variations of six bee viruses in *Apis mellifera* L. and *Varroa destructor* mite populations in France. Appl Environ Microbiol 70, 7185–7191 10.1128/AEM.70.12.7185-7191.2004 15574916 PMC535170

[r65] Tentcheva D. Gauthier L. Jouve S. Canabady-Rochelle L. Dainat B. Cousserans F. Colin M. E. Ball B. V. Bergoin M. **(**2004b**).** Polymerase chain reaction detection of deformed wing virus (DWV) in *Apis mellifera* and *Varroa destructor*. Apidologie (Celle) 35, 431–439 10.1051/apido:2004021

[r66] Todd J. H. Ball B. V. de Miranda J. R. **(**2004**).** Identifying the viruses causing mortality of honey bees in colonies infested with *Varroa destructor*. Surveillance 31, 22–25

[r67] Todd J. H. De Miranda J. R. Ball B. V. **(**2007**).** Incidence and molecular characterization of viruses found in dying New Zealand honey bee (*Apis mellifera*) colonies infested with *Varroa destructor*. Apidologie (Celle) 38, 354–367 10.1051/apido:2007021

[r68] Vanengelsdorp D. Evans J. D. Saegerman C. Mullin C. Haubruge E. Nguyen B. K. Frazier M. Frazier J. Cox-Foster D. **& other authors (**2009**).** Colony collapse disorder: a descriptive study. PLoS ONE 4, e6481 10.1371/journal.pone.0006481 19649264 PMC2715894

[r69] Visscher P. K. **(**1989**).** A quantitative study of worker reproduction in honey bee colonies. Behav Ecol Sociobiol 25, 247–254 10.1007/BF00300050

[r70] Williams G. R. Rogers R. E. L. Kalkstein A. L. Taylor B. A. Shutler D. Ostiguy N. **(**2009**).** Deformed wing virus in western honey bees (*Apis mellifera*) from Atlantic Canada and the first description of an overtly-infected emerging queen. J Invertebr Pathol 101, 77–79 10.1016/j.jip.2009.01.004 19373971

[r71] Winston M. L. **(**1987**).** The Biology of the Honey Bee. Cambridge, MA: Harvard University Press

[r72] Yanez O. Jaffe R. Jarosch A. Fries I. Moritz R. F. A. Paxton R. J. de Miranda J. R. **(**2012**).** Deformed wing virus and drone mating flights in the honey bee (*Apis* *mellifera*): implications for sexual transmission of a major honey bee virus. Apidologie (Celle) 43, 17–30 10.1007/s13592-011-0088-7

[r73] Yue C. Genersch E. **(**2005**).** RT-PCR analysis of *Deformed wing virus* in honeybees (*Apis mellifera*) and mites (*Varroa destructor*). J Gen Virol 86, 3419–3424 10.1099/vir.0.81401-0 16298989

[r74] Yue C. Schröder M. Bienefeld K. Genersch E. **(**2006**).** Detection of viral sequences in semen of honeybees (*Apis mellifera*): evidence for vertical transmission of viruses through drones. J Invertebr Pathol 92, 105–108 10.1016/j.jip.2006.03.001 16630626

[r75] Yue C. Schröder M. Gisder S. Genersch E. **(**2007**).** Vertical-transmission routes for deformed wing virus of honeybees (*Apis mellifera*). J Gen Virol 88, 2329–2336 10.1099/vir.0.83101-0 17622639

[r76] Zhang Q. Ongus J. R. Boot W. J. Calis J. Bonmatin J.-M. Bengsch E. Peters D. **(**2007**).** Detection and localisation of picorna-like virus particles in tissues of *Varroa* *destructor*, an ectoparasite of the honey bee, *Apis mellifera*. J Invertebr Pathol 96, 97–105 10.1016/j.jip.2007.03.019 17574570

